# Vitellogenin Facilitates Associations between the Whitefly and a Bacteriocyte Symbiont

**DOI:** 10.1128/mbio.02990-22

**Published:** 2023-01-24

**Authors:** Xiang Sun, Bing-Qi Liu, Zhan-Bo Chen, Chu-Qiao Li, Xing-Ye Li, Ji-Sheng Hong, Jun-Bo Luan

**Affiliations:** a Liaoning Key Laboratory of Economic and Applied Entomology, College of Plant Protection, Shenyang Agricultural University, Shenyang, China; National Institute of Advanced Industrial Science and Technology (AIST); University of Hawaii at Manoa

**Keywords:** bacteriocyte, juvenile hormone, symbiont, transmission, vitellogenin, whitefly

## Abstract

Integration between animal reproduction and symbiont inheritance is fundamental in symbiosis biology, but the underlying molecular mechanisms are largely unknown. Vitellogenin (Vg) is critical for oogenesis, and it is also a pathogen pattern recognition molecule in some animals. Previous studies have shown that Vg is involved in the regulation of symbiont abundance and transmission. However, the mechanisms by which an insect and its symbiont contribute to the function of Vg and how Vg impacts the persistence of insect-microbe symbiosis remain largely unclear. Symbionts are transovarially transmitted via maternal inheritance of the bacteriocytes in the whitefly Bemisia tabaci. Surprisingly, Vg is localized in bacteriocytes of whiteflies. Vg could be synthesized in whitefly bacteriocytes by the gene *Vg* expressed in these cells or exported into bacteriocytes from hemolymph via the Vg receptor. We further found that the juvenile hormone and “*Candidatus* Portiera aleyrodidarum” (here termed *Portiera*) control the level and localization of Vg in whiteflies. Immunocapture PCR revealed interactions between Vg and *Portiera*. Suppressing Vg expression reduced *Portiera* abundance as well as whitefly oogenesis and fecundity. Thus, we reveal that Vg facilitated the persistence of whitefly-bacteriocyte symbiont associations. This study will provide insight into the key role of Vg in the coevolution of insect reproduction and symbiont inheritance.

## INTRODUCTION

Heritable symbionts are ubiquitous in nature, and symbiotic relationships have driven the ecological and evolutionary diversification of animals (1, 2). Inherited symbionts include obligate symbionts that are required by the host and facultative symbionts that are not essential to hosts but that may confer important traits (1, 2). Inherited symbionts affect animal reproduction in many ways (3–6). For example, several obligate symbionts influence the fecundity or sex ratio of their hosts by providing specific nutrients (7–13). These symbionts can promote their own vertical transmission by affecting animal reproduction (7–17). The proliferation and inheritance of symbionts have been integrated into the reproduction and development of animals (3–6, 17). Successful reproduction of animals and transmission of symbionts thus require coadaptation between animals and symbionts. However, the physiological, biochemical, and molecular mechanisms underlying the coordination of animal reproduction and symbiont transmission are largely unknown.

Vitellogenin (Vg) is a large glycolipophosphoprotein and a precursor of the major egg yolk protein vitellin in many oviparous animals (18). The proteins are transported to the ovaries and incorporated into developing oocytes via Vg receptor (VgR)-mediated endocytosis (18). Vg is critical for oogenesis because it provides essential nutrients such as amino acids, lipids, and vitamins (18–20). A common form of vertical inheritance is transovarial transmission, which is typical for hereditary symbionts (3, 6, 21–24). Some symbionts use the host’s molecular and cellular machinery for targeting oocytes (23, 24). For example, *Spiroplasma* in *Drosophila* interacts with Vg and migrates to oocytes by coopting the VgR-mediated endocytotic mechanism (24). Vg is also a pathogen pattern recognition molecule in some animals. Vg is capable of killing or binding bacteria via interaction with lipopolysaccharide and lipoteichoic acid, as in fish, or by binding to peptidoglycan, lipopolysaccharide, and zymosan, as in the honey bee Apis mellifera (25, 26). Vg acts as an antimicrobial agent in the Asian honey bee, Apis cerana (27), and it is required for transgenerational immunity in *A. mellifera* (26). *Vg* expression influences the levels of *Rickettsia* in whiteflies and *Wolbachia* in planthoppers (28, 29). Vg appears to play a key role in insect-symbiont interactions. However, the mechanisms by which insect symbionts regulate the function of Vg and how Vg impacts the persistence of insect-microbe symbiosis remain largely unclear.

Whitefly symbiosis is a useful model system to study the persistence of insect-microbe symbiosis. The whitefly Bemisia tabaci is a complex of more than 40 cryptic species (30), among which *B. tabaci* MEAM1 is an invasive and globally important agricultural pest (31). All *B. tabaci* species harbor the obligate symbiont “*Candidatus* Portiera aleyrodidarum” (here termed *Portiera*) in specialized insect cells called bacteriocytes, and they harbor up to four facultative symbiont lineages (32, 33). In China, *B. tabaci* MEAM1 bears *Portiera* and “*Candidatus* Hamiltonella defensa” (here termed *Hamiltonella*) in the same bacteriocyte (34, 35). *Portiera* and *Hamiltonella* are transovarially transmitted via maternal inheritance of the bacteriocytes (21, 36). *Portiera* is involved in the synthesis of 10 essential amino acids (EAAs) (37, 38), which may influence protein synthesis. Additionally, the juvenile hormone (JH) regulates the Vg level in many insects (39–41). Thus, we proposed that JH and *Portiera* may regulate the Vg level in whiteflies and that Vg could impact whitefly reproduction and symbiont persistence. To test this hypothesis, we examined the localization of Vg in both ovarioles and bacteriocytes of whiteflies, studied how whitefly JH and *Portiera* affect the level and localization of Vg, and investigated how Vg impacts the whitefly and bacteriocyte symbiont associations. We found that Vg is localized in both ovarioles and bacteriocytes of whiteflies, that whitefly JH and *Portiera* positively adjust the level and localization Vg in whiteflies, and that Vg affects whitefly reproduction and the abundance and transmission of bacteriocyte symbionts.

## RESULTS

### Vg is localized in bacteriocytes of whiteflies.

**FIG 1:**
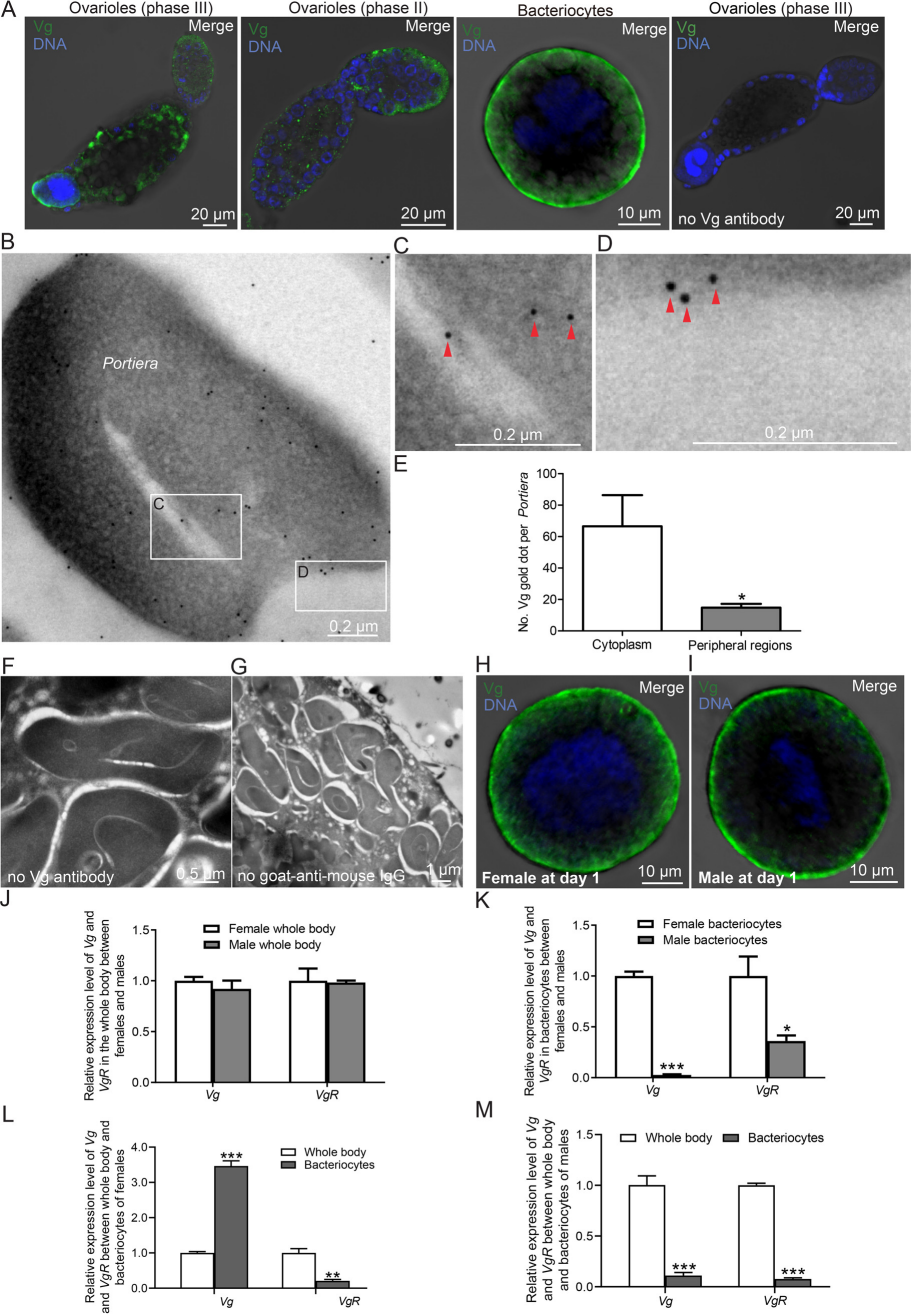
Vg localization in whitefly bacteriocytes. (A) Localization of Vg (green) in ovarioles and bacteriocytes of ovarioles at mid-vitellogenesis phase III, in ovarioles at early vitellogenesis phase II, and in bacteriocytes of female adult whiteflies at days 1 to 6 after emergence. Ovarioles at mid-vitellogenesis phase III of female adult whiteflies were incubated with no antibodies against Vg as the negative control. (B to E) Immunoelectron micrograph showing the presence of Vg within the cytoplasm (B and C) of *Portiera* and on the peripheral regions (B and D) of *Portiera*, which could be the cell membrane of *Portiera* or the insect symbiosome membrane, in bacteriocytes. The bacteriocytes were immunolabeled with Vg-specific IgG as the primary antibody, followed by treatment with 10-nm gold particle-conjugated goat antibodies against mouse IgG as the secondary antibody. Panels C and D are enlargements of the boxed areas in panel B. The red arrowheads indicate gold particles. (E) Number of Vg gold dots per *Portiera*. (F and G) Immunoelectron micrographs showing the absence of Vg in *Portiera* in bacteriocyte sections of the negative controls. The samples were treated without primary antibody (F) or without gold-conjugated goat anti-mouse IgG (G) as the controls. (H and I) Localization of Vg (green) in bacteriocytes of female (H) and male (I) adult whiteflies at day 1 after emergence. (J) Expression of *Vg* and *VgR* in the whole body of female and male adult whiteflies at day 1 after emergence. (K) Expression of *Vg* and *VgR* in bacteriocytes of female and male adult whiteflies at day 1 after emergence. (L and M) Relative expression levels of *Vg* and *VgR* between whole body and bacteriocytes of female (L) and male (M) adult whiteflies at day 1 after emergence. Data are means ± standard error (SE). *n* = 4, 3, 3, 3, and 3 for panels E, J, K, L, and M. DNA was stained with DAPI (4′,6-diamidino-2-phenylindole) in panels A, H, and I. All of the images are representative of three replicates for immunofluorescence microscopy and four replicates for immunoelectron microscopy. Significant differences between treatments are indicated by asterisks in panels E and K–M (*, *P < *0.05; **, *P < *0.01; ***, *P < *0.001).

We examined the subcellular location of the whitefly Vg protein in ovaries by using a previously produced monoclonal antibody against Vg protein (42). Immunofluorescence microscopy showed that Vg was expressed in whitefly ovarioles at vitellogenesis phase III and their associated bacteriocytes (Fig. 1A). Vg was located mainly in the follicle cells, nurse cells, and oocytes of ovarioles at vitellogenesis phase II (Fig. 1A) as well as the cytoplasmic regions near the cell surfaces in contact with the external medium of bacteriocytes (Fig. 1A). The absence of a signal in the negative controls without primary antibody confirmed the specificity of Vg in whitefly bacteriocytes (Fig. 1A).

A parallel immunoelectron microscopy assay confirmed that Vg was present within the cytoplasm of *Portiera* (Fig. 1B and C) and on the peripheral regions of *Portiera* (Fig. 1B and D), which could be the cell membrane of *Portiera* or the insect symbiosome membrane in the bacteriocytes. There were more Vg gold dots within the cytoplasm of *Portiera* than on the peripheral regions of *Portiera* (Fig. 1E; *P = *0.042). Vg was absent within the cytoplasm or on the peripheral regions of *Portiera* (Fig. 1F and G) when bacteriocyte sections were treated without primary antibody or gold-conjugated goat-anti-mouse IgG as the controls.

Vg is synthesized mainly in the fat body, moves through the hemolymph, and is transported from the hemolymph to oocytes via VgR present on the surface of oocytes (43, 44). The bacteriocyte is vertically transmitted by adult female whiteflies, which have ovarioles, but not by adult males, which have no ovarioles (45). Immunofluorescence microscopy showed that Vg was highly expressed in bacteriocytes of female whiteflies compared to that in male whiteflies (Fig. 1H and I). At this point, it is not clear whether Vg protein is inherently expressed or simply exported and present in the bacteriocytes. We further detected the expression of *Vg* and *VgR* in the whole body and bacteriocytes of female and male whiteflies. The expression level of *Vg* or *VgR* is similar in the whole body of female and male whiteflies at day 1 after emergence (Fig. 1J). In contrast, the expression level of *Vg* or *VgR* is significantly higher in bacteriocytes of female whiteflies than in male whiteflies at day 1 after emergence (Fig. 1K). The expression level of *Vg* is significantly higher, but the expression level of *VgR* is significantly lower, in bacteriocytes than in the whole body of female whiteflies (Fig. 1L). The expression level of *Vg* or *VgR* is significantly lower in bacteriocytes than in the whole body of male whiteflies (Fig. 1M). These results suggest that Vg in whitefly bacteriocytes is produced either by the gene *Vg* expressed in whitefly bacteriocytes or by protein Vg exportation from the hemolymph via *VgR* in both females and males.

### JH controls Vg expression and localization in whiteflies.

**FIG 2:**
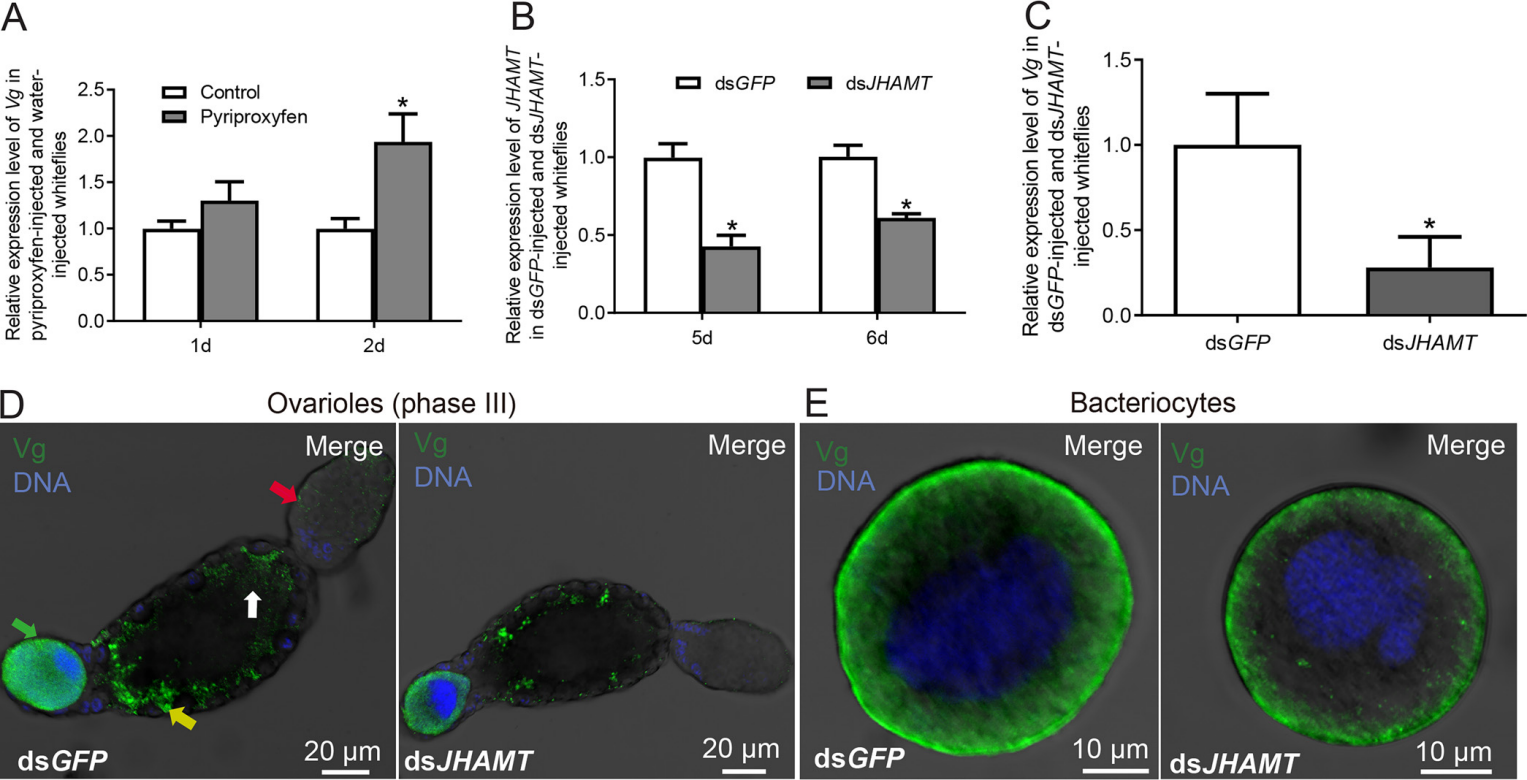
JH controls Vg expression and localization. (A) Expression of *Vg* in female adult whiteflies at days 1 and 2 after whiteflies were microinjected with the JH analog pyriproxyfen at 0.5 μg/μL. Distilled-water-injected whiteflies were used as the control. (B) Expression of *JHAMT* in ds*GFP*-injected and ds*JHAMT*-injected female adult whiteflies at days 5 and 6 after whiteflies were microinjected with dsRNA. (C) Expression of *Vg* in ds*GFP*-injected and ds*JHAMT*-injected female adult whiteflies at day 5 after whiteflies were microinjected with dsRNA. (D) Localization of Vg (green) in ovarioles at mid-vitellogenesis phase III of ds*GFP*-injected and ds*JHAMT*-injected female adult whiteflies at day 5 after whiteflies were microinjected with dsRNA. The yellow, red, white, and green arrows denote localization of Vg in the follicle cells, nurse cells, oocytes, and bacteriocytes, respectively, of ovaries. (E) Localization of Vg (green) in bacteriocytes of ds*GFP*-injected and ds*JHAMT*-injected female adult whiteflies at day 5 after whiteflies were microinjected with dsRNA. DNA was stained with DAPI in panels D and E. All of the images are representative of three replicates. Data are means ± SE. *n* = 3 in panels A to C. Significant differences between treatments are indicated by asterisks in panels A to C (*, *P < *0.05).

JH regulates the Vg level in many insects (39–41). To test whether JH impacts *Vg* expression and Vg localization, whiteflies were injected with the JH analog pyriproxyfen. Expression of *Vg* was significantly upregulated at day 2 after injection in the whole body of pyriproxyfen-injected wild-type whiteflies compared to that in control whiteflies (Fig. 2A; *P* = 0.24 and 0.044 for day 1 and day 2, respectively). JH acid methyltransferase (JHAMT) is a key enzyme that converts JH acids or inactive precursors of JHs to active JHs during the final step of the JH synthesis pathway in insects (46). To test the effect of JH on *Vg* expression and Vg localization, whitefly *JHAMT* was silenced. *JHAMT* expression was reduced by 57% and 39% at 5 and 6 days, respectively, after microinjection with double-stranded RNAs (dsRNAs) (Fig. 2B; *P* = 0.0079 and 0.0075). Expression of *Vg* was significantly downregulated in ds*JHAMT* (dsRNA specific to whitefly *JHAMT*)-injected whiteflies compared to that in ds*GFP* (dsRNA specific to *GFP*)-injected whiteflies (Fig. 2C; *P* = 0.023) at day 5 postinjection. To test whether silencing *JHAMT* influenced expression of Vg, we examined the subcellular location of the whitefly Vg protein in ovaries and bacteriocytes by using the monoclonal antibody against Vg protein (42). Immunofluorescence microscopy showed that Vg localization declined in both ovarioles and bacteriocytes of ovarioles at vitellogenesis phase III as well as in bacteriocytes of ds*JHAMT*-injected whiteflies compared to that in ds*GFP*-injected whiteflies at day 5 after microinjection (Fig. 2D and E and see Fig. S1 in the supplemental material; *P* = 0.0033 and *P* = 0.011).

10.1128/mbio.02990-22.1 FIG S1 Effect of *JHAMT* silencing on fluorescence intensity of Vg in whitefly ovarioles and bacteriocytes. Whitefly ovarioles at mid-vitellogenesis phase III and bacteriocytes of ds*GFP*-injected and ds*JHAMT*-injected female adult whiteflies at day 5 after whiteflies were microinjected with dsRNA were used for analysis. Data are means ± SE. *n* = 3. Significant differences between treatments are indicated by asterisks (*, *P < *0.05; **, *P < *0.01). Download FIG S1, TIF file, 0.05 MB. Copyright © 2023 Sun et al. 2023 Sun et al. https://creativecommons.org/licenses/by/4.0/ This content is distributed under the terms of the Creative Commons Attribution 4.0 International license.

### *Portiera* elimination represses whitefly oogenesis and Vg localization.

**FIG 3:**
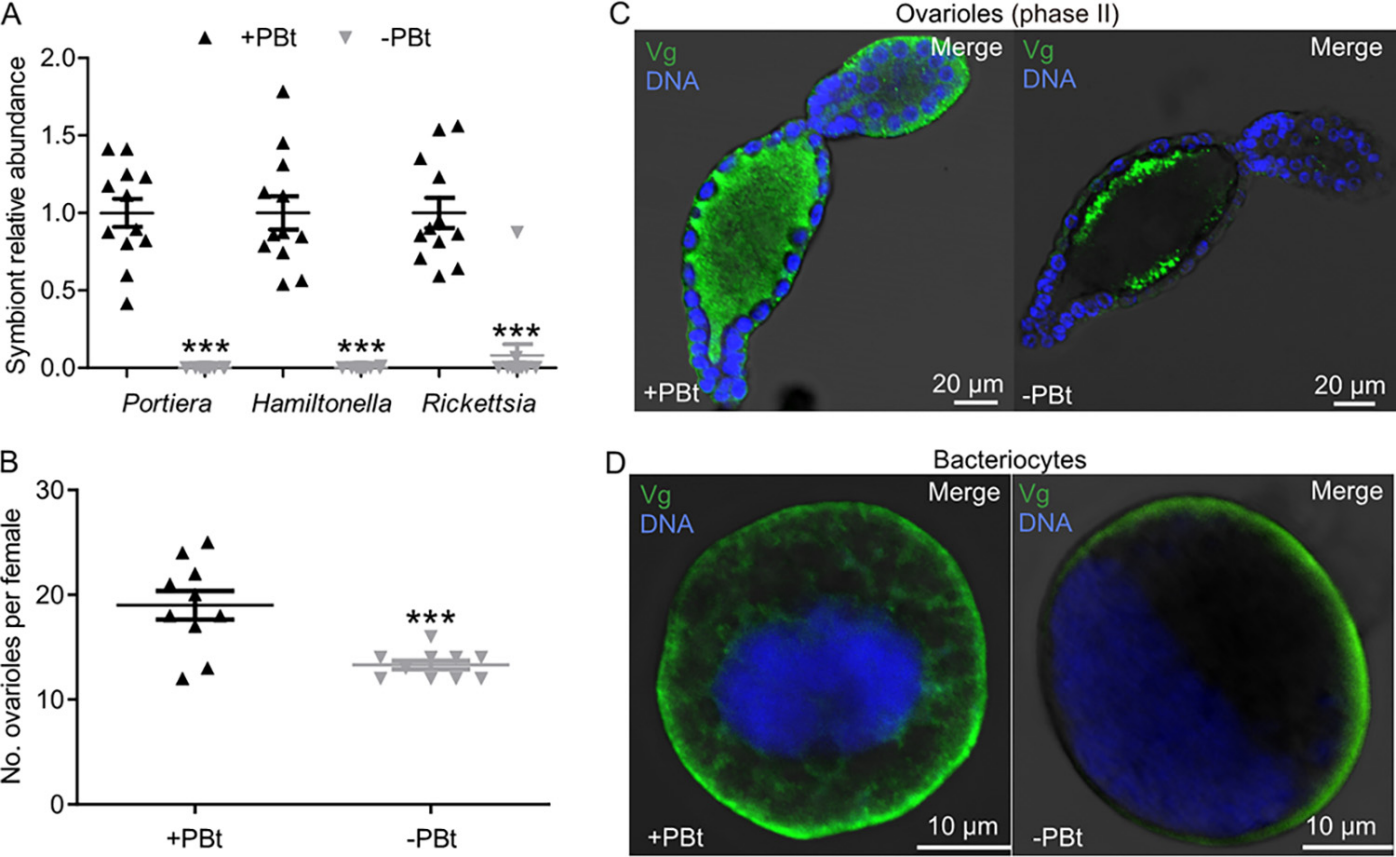
Effects of *Portiera* elimination on whitefly oogenesis and Vg localization. (A) Effects of antibiotic treatments on the abundance of symbionts in *B. tabaci*. (B) The number of ovarioles of +PBt and −PBt female adult whiteflies within 4 days after emergence. (C and D) Localization of Vg (green) in ovarioles at early vitellogenesis phase II (C) and in bacteriocytes (D) of +PBt and −PBt female adult whiteflies. DNA was stained with DAPI. Data are means ± SE. *n* = 12 and 10 in panels A and B, respectively. All images are representative of three replicates. Significant differences between treatments are indicated by asterisks in panels A and B (***, *P < *0.001).

*Portiera* elimination can reduce whitefly fecundity (14). Thus, the total number of ovarioles and Vg localization in ovarioles and bacteriocytes were examined in *Portiera*-infected F1 *B. tabaci* (+PBt) and *Portiera*-cured F1 *B. tabaci* (−PBt) female whiteflies. The *Portiera* abundance was reduced by 99.9%, and the abundance of *Hamiltonella* and *Rickettsia* was reduced by 99.8% and 91.9%, respectively, by rifampin treatment (Fig. 3A; *P < *0.0001). The total number of ovarioles was decreased significantly in −PBt whiteflies compared to that in +PBt whiteflies (Fig. 3B, *P = *0.00083; Fig. S2A). After *Portiera* was cured, Vg localization was repressed in both ovarioles and bacteriocytes of whiteflies (Fig. 3C and D and Fig. S2B; *P* = 0.014 and *P* = 0.0032). These data suggest that the abundance of *Portiera* impacts whitefly oogenesis and Vg localization in whitefly ovarioles and bacteriocytes.

10.1128/mbio.02990-22.2 FIG S2 Effects of *Portiera* elimination on whitefly ovaries and fluorescence intensity of Vg in whitefly ovarioles and bacteriocytes. Download FIG S2, TIF file, 1.1 MB. Copyright © 2023 Sun et al. 2023 Sun et al. https://creativecommons.org/licenses/by/4.0/ This content is distributed under the terms of the Creative Commons Attribution 4.0 International license.

### Repressing Vg reduces symbiont abundance and whitefly reproduction.

**FIG 4:**
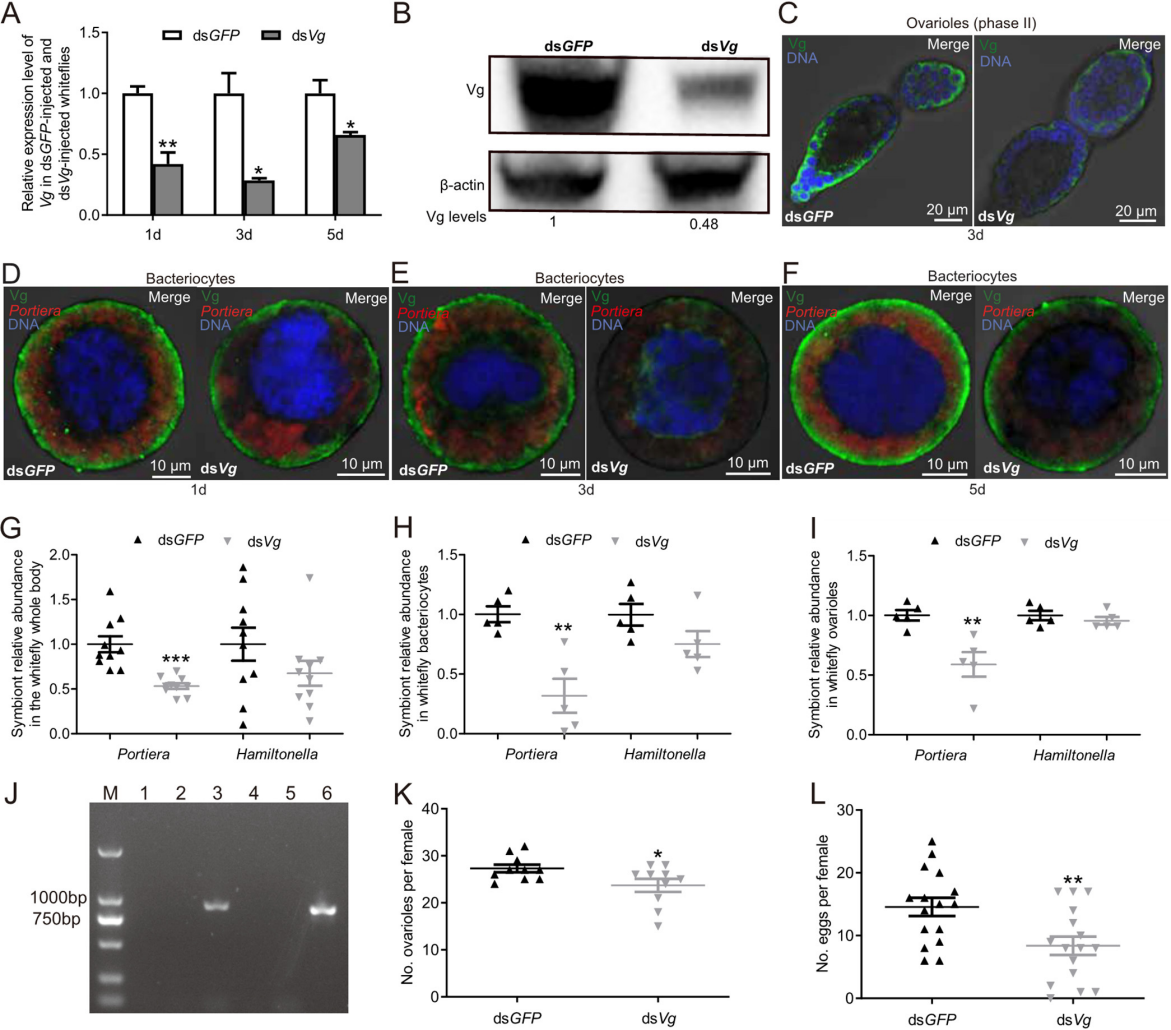
Repressing Vg reduces symbiont abundance and whitefly reproduction. (A) Expression of *Vg* in ds*GFP*-injected and ds*Vg*-injected female adult whiteflies at 1, 3, and 5 days after whiteflies were microinjected with dsRNA. (B) Western blot analysis of Vg levels in ds*Vg*-injected and ds*GFP*-injected whiteflies at day 3 after whiteflies were microinjected with dsRNA. β-Actin is shown as a loading control. The level of Vg protein in whiteflies is normalized to β-actin and indicated beneath the blots. The level of Vg protein indicates the mean of three biological replicates. (C) Localization of Vg (green) in ovarioles of ds*GFP*-injected and ds*Vg*-injected female adult whiteflies at day 3 after whiteflies were microinjected with dsRNA. (D to F) Localization of Vg (green) and *Portiera* (red) in bacteriocytes of ds*GFP*-injected and ds*Vg*-injected female adult whiteflies at days 1, 3, and 5 after whiteflies were microinjected with dsRNA. (G to I) The symbiont abundance in the whole body (G), bacteriocytes (H), and ovarioles (I) of whiteflies at day 3 after whiteflies were microinjected with ds*Vg*. (J) Immunodetection of vitellogenin-*Portiera* interactions. Antibody-coated tubes and *Portiera*-specific primers were used to detect the interaction. Lane M, 2,000-bp ladder marker; lanes 1 and 2, *Portiera*-infected (bacteriocytes) and uninfected (heads) extracts, respectively, with no-antibody controls; lanes 3 and 4, *Portiera*-infected (bacteriocytes) and uninfected (heads) extracts, respectively, with Vg antibody; lane 5, no-template control; lane 6, control PCR for *Portiera.* (K) The number of ovarioles per female at day 3 after whiteflies were microinjected with ds*Vg*. (L) Female fecundity at day 3 after whiteflies were microinjected with ds*Vg*. Data are means ± SE. *n* = 3, 10, 5, 5, 15, and 10 in panels A, G, H, I, K, and L. DNA was stained with DAPI in panels (C–F). All of the images are representative of three replicates. Significant differences between treatments are indicated by asterisks in panels A, G, H, I, K, and L (*, *P < *0.05; **, *P < *0.01; ***, *P < *0.001).

Vg is indispensable for insect reproduction and also functions as a pathogen pattern recognition molecule in some animals (18, 25, 26). To investigate the effect of repressing Vg on the abundance of the two symbionts in bacteriocytes and on whitefly reproduction, whitefly *Vg* was silenced by microinjection with ds*Vg* (dsRNA specific to whitefly *Vg*). Vg localization in ovarioles and bacteriocytes, the abundance of the two symbionts, and the numbers of ovarioles and eggs were then examined. Expression of whitefly *Vg* was reduced by 58%, 72%, and 34% at 1, 3, and 5 days, respectively, after ds*Vg* injection (Fig. 4A; *P < *0.05). Western blot analysis showed that the Vg level was significantly decreased in ds*Vg*-injected whiteflies compared with that in ds*GFP*-injected whiteflies (Fig. 4B; *P = *0.0013). As a result of the gene silencing, Vg localization was noticeably reduced in ovarioles and bacteriocytes at days 1, 3, and 5 after RNA interference (RNAi) treatment (Fig. 4C to F; Fig. S3A and B and S4A; *P < *0.01 for ovarioles and *P < *0.05 for bacteriocytes). FISH (fluorescence *in situ* hybridization) observation showed that the abundance of *Portiera* was decreased in bacteriocytes (Fig. 4D to F; Fig. S4B; *P < *0.01), and *Hamiltonella* distribution remained unchanged in bacteriocytes (Fig. S5). A quantitative PCR (qPCR) test also showed that the abundance of *Portiera* was significantly decreased in the whole body, bacteriocytes, and ovaries of whiteflies at day 3 after *Vg* RNAi treatment (Fig. 4G to I; *P = *0.00012, *P = *0.0025, and *P = *0.0059, respectively). In contrast, the abundance of *Hamiltonella* was not significantly reduced in the whole body, bacteriocytes, and ovaries of whiteflies at day 3 after *Vg* RNAi treatment (Fig. 4G to I; *P = *0.18, *P = *0.11, and *P = *0.37, respectively). To examine the interaction between Vg and *Portiera*, immunocapture PCR (IC-PCR) was conducted using an anti-Vg antibody and *Portiera*-specific 16S rRNA gene fragment primers. IC-PCR suggested the interactions between Vg and *Portiera* (Fig. 4J). In parallel with the changes in Vg localization, the number of ovarioles and the number of eggs were significantly decreased in ds*Vg*-injected whiteflies compared to those in ds*GFP*-injected whiteflies (Fig. 4K and L; *P = *0.038 and 0.0055, respectively).

10.1128/mbio.02990-22.3 FIG S3 Effect of *Vg* silencing on localization and fluorescence intensity of Vg in whitefly ovarioles. (A) Localization of Vg (green) in ovarioles of ds*GFP*-injected and ds*Vg*-injected female adult whiteflies at 1 and 5 days after whiteflies were microinjected with dsRNA. DNA was stained with DAPI. All of the images are representative of three replicates. (B) Fluorescence intensity of Vg in ovarioles of ds*GFP*-injected and ds*Vg*-injected female adult whiteflies at 1, 3, and 5 days after whiteflies were microinjected with dsRNA. Data are means ± SE. *n* = 3. Significant differences between treatments are indicated by asterisks (**, *P < *0.01; ***, *P < *0.001). Download FIG S3, TIF file, 0.6 MB. Copyright © 2023 Sun et al. 2023 Sun et al. https://creativecommons.org/licenses/by/4.0/ This content is distributed under the terms of the Creative Commons Attribution 4.0 International license.

10.1128/mbio.02990-22.4 FIG S4 Effect of *Vg* silencing on fluorescence intensity of Vg and *Portiera* in whitefly bacteriocytes. Fluorescence intensity of Vg (A) and *Portiera* (B) in bacteriocytes of ds*GFP*-injected and ds*Vg*-injected female adult whiteflies at 1, 3, and 5 days after whiteflies were microinjected with dsRNA. Data are means ± SE. *n* = 3. Significant differences between treatments are indicated by asterisks (*, *P < *0.05; **, *P < *0.01; ***, *P < *0.001). Download FIG S4, TIF file, 0.1 MB. Copyright © 2023 Sun et al. 2023 Sun et al. https://creativecommons.org/licenses/by/4.0/ This content is distributed under the terms of the Creative Commons Attribution 4.0 International license.

10.1128/mbio.02990-22.5 FIG S5 Effect of *Vg* silencing on distribution of *Hamiltonella* in whitefly bacteriocytes. Localization of *Portiera* (red) and *Hamiltonella* (green) in bacteriocytes of ds*GFP*-injected (A) and ds*Vg*-injected (B) female adult whiteflies at day 3 after whiteflies were microinjected with dsRNA. DNA was stained with DAPI. All of the images are representative of three replicates. Download FIG S5, TIF file, 0.5 MB. Copyright © 2023 Sun et al. 2023 Sun et al. https://creativecommons.org/licenses/by/4.0/ This content is distributed under the terms of the Creative Commons Attribution 4.0 International license.

## DISCUSSION

**FIG 5:**
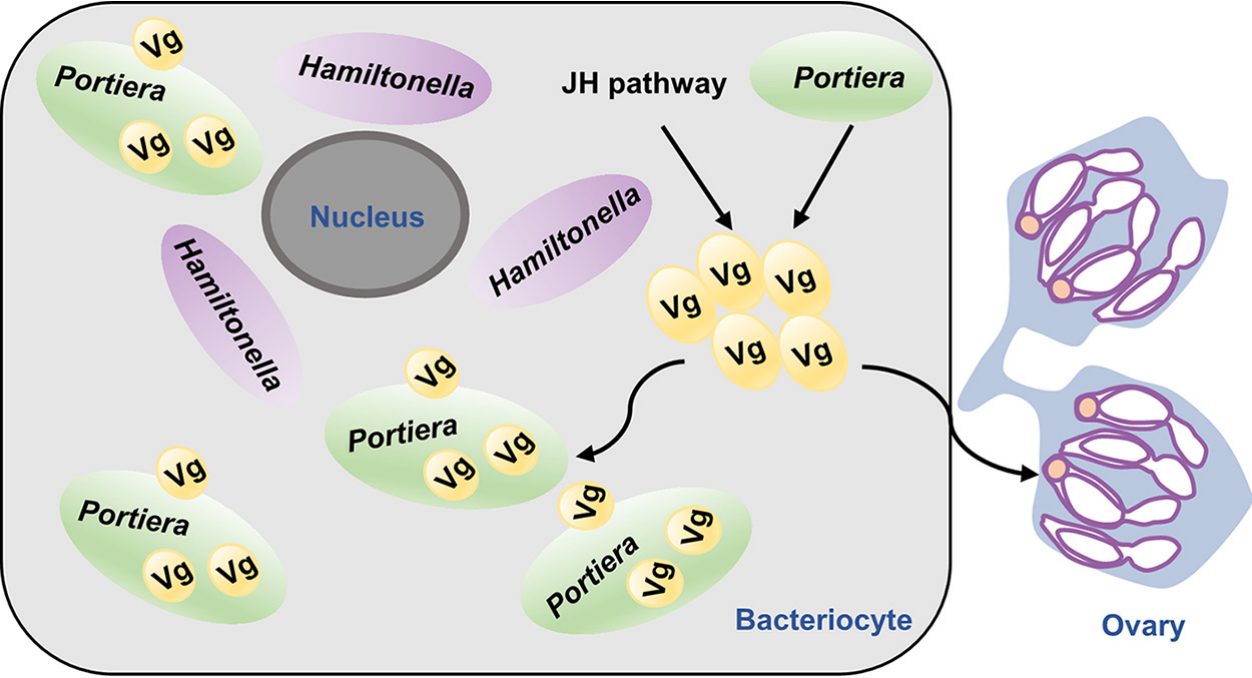
Schematic overview of how vitellogenin (Vg) contributes to the persistence of whitefly symbiosis. The juvenile hormone (JH) and symbiont *Portiera* impact Vg localization in whiteflies. Vg facilitates the abundance and transmission of bacteriocyte symbionts as well as whitefly oogenesis and fecundity.

The integration of insect reproduction and symbiont inheritance is critical for the persistence of insect-microbe symbiosis. However, the molecular mechanisms underlying the integration and coevolution of animal reproduction and symbiont inheritance are largely unknown. In this study, we found that JH and *Portiera* upregulate the level and localization of Vg in whitefly ovaries and bacteriocytes, and in turn, Vg facilitates the abundance and transmission of *Portiera* and whitefly oogenesis and fecundity (Fig. 5). This study reveals that the multifunctional protein Vg has become the central player in the integration between symbiont inheritance and insect reproduction or development. Our data also indicate that the bacteriocyte’s function with Vg expression is the key for whitefly symbiosis evolution. Although this interaction of Vg and symbionts should still be treated with caution to be considered universal, our results provide insight into the coevolution of insect reproduction and symbiont inheritance.

Generally, Vg is synthesized mainly in the fat body and transported to growing oocytes by membrane-bound VgR through receptor-mediated endocytosis (43, 44). Surprisingly, we found that Vg is also present in whitefly bacteriocytes. Vg in whitefly bacteriocytes can be generated by the gene *Vg* expressed in whitefly bacteriocytes and exported from the hemolymph via *VgR* expressed in whitefly bacteriocytes. The expression of the genes *Vg* and *VgR* as well as the protein Vg in whitefly bacteriocytes is unique in insect symbiosis.

JH can control the Vg level in whiteflies as in most hemipterans by regulating *Vg* gene transcription (40, 41). In contrast, *Portiera* determines the level and localization of Vg in female adult whiteflies by directly influencing protein synthesis, as this symbiont can provide multiple EAAs. *Portiera* elimination decreases whitefly fecundity (13). Our previous work demonstrated that *Portiera* can cooperate with horizontally transferred genes of whiteflies for the synthesis of lysine, which impacts whitefly fecundity (47). Vg is a precursor of the major egg yolk protein (18). Thus, *Portiera* can affect Vg synthesis in female adult whiteflies by providing EAAs, thereby influencing whitefly fecundity. Other bacteriocyte symbionts can also synthesize pantothenate or EAAs (2, 48, 49), thus influencing Vg levels in insects, too. Therefore, it could be common that host symbiosis impacts the Vg level in insects harboring bacteriocyte-associated symbionts.

Vg can provide critical nutrients for oogenesis and embryogenesis of insects, and it is also a pathogen pattern recognition molecule in some animals. *Rice stripe virus* and *Tomato yellow leaf curl virus* can bind to Vg for transovarial transmission in small brown planthoppers and whiteflies, respectively (50, 51). The symbiont *Spiroplasma* uses the host Vg transovarial transportation system for vertical transmission in *Drosophila* (24), and the symbiont *Nasuia* interacts with Vg during vertical transmission in leafhoppers (52). We found that Vg was present within the cytoplasm and on the peripheral regions of *Portiera*. Repressing Vg reduced the abundance of *Portiera* in bacteriocytes. IC-PCR revealed that there are interactions between Vg and *Portiera*. Overall, the presence of Vg in the cytoplasm of the symbiont and the regulation of symbiont abundance by Vg indicate that Vg may have the function of the pathogen pattern recognition molecule as reported in fish and honeybees (53–55). The novel function of Vg facilitates the interaction between Vg and symbiont.

Although *Portiera* and *Hamiltonella* colocalize in the same bacteriocyte, our study showed that disruption of *Vg* negatively impacted *Portiera* localization in bacteriocytes but had no effect on *Hamiltonella*. That could be caused by the localization of Vg and symbionts in the bacteriocyte and the differential cell structure of *Portiera* and *Hamiltonella*. Vg was located mainly in the cytoplasmic regions near the cell surfaces in contact with the external medium of bacteriocytes (Fig. 1A, H, and I, 2E, 3D, and 4D to F). *Portiera* occupied the cytoplasmic regions of bacteriocytes, while *Hamiltonella* was distributed mainly around bacteriocyte nuclei (34, 36, 45). Thus, the localization of Vg in the bacteriocyte is closer to that of *Portiera* than to *Hamiltonella* in the bacteriocyte. Additionally, *Portiera* is more ancient than *Hamiltonella*, so *Portiera* has a highly reduced genome and has lost its cell wall (36, 56, 57). The interactions between Vg and *Portiera* could facilitate the persistence of this symbiont in bacteriocytes. In contrast, *Hamiltonella* is almost surrounded by *Portiera* and has an intact cell wall. *Hamiltonella* may not need the help of Vg for its maintenance in bacteriocytes. Overall, it seems that *Hamiltonella* uses a different Vg-independent mechanism. Therefore, the Vg symbiosis elucidated in the study is specific only to *Portiera*. Additionally, there was a higher expression level of Vg in bacteriocytes of female whiteflies than in bacteriocytes of male whiteflies. The effect of Vg on host biology and symbiont abundance in male whiteflies remains unknown. Thus, whether *Portiera* abundance in the whitefly is governed by a general regulatory mechanism should be tested in males or younger nymphs of whiteflies in future studies.

In this study, we refer to the relative symbiont abundance or density between various treatments. Both the aphid and *Buchnera* experience polyploidy, which dramatically changes throughout insect development for both players (58, 59). Whitefly bacteriocytes and associated symbionts may also show polyploidy as in the aphid-*Buchnera* system. What have actually been measured here are genome copies of the host and symbiont. Perhaps it would be more conservative to call this symbiont genome copies relative to the insect genome copies.

Some studies have shown that Vg delivery for oocyte maturation in the host is coupled with vertical transmission of the symbiont (24, 52). We demonstrated that the whitefly and symbiont impact the levels and localization of Vg in bacteriocytes and ovarioles. When the expression of Vg in bacteriocytes and ovarioles was repressed, there was insufficient Vg protein binding to symbiont so that *Portiera* could be degraded by the immune response. Therefore, silencing Vg reduced the abundance of *Portiera* in the bacteriocytes and ovarioles of female adult whiteflies. *Portiera* and *Hamiltonella* are transmitted into ovarioles via bacteriocyte transfer in whiteflies (21, 36). The development of both ovarioles and bacteriocytes impacts the vertical transmission of *Portiera* in whiteflies. Repressing Vg decreased the number of whitefly ovarioles and eggs, thus inhibiting the transovarial transmission of symbionts. Overall, Vg influences both the abundance and transmission of *Portiera* in female adult whiteflies. Vg, which is indispensable for oogenesis, seems to gain a novel function involved in symbiont maintenance during long-term evolution of whitefly symbiosis. This study of whitefly symbiosis indicates that Vg is a key player in insect-symbiont interactions and coevolution. It will be interesting to study how Vg impacts the persistence of various insect-microbe symbioses. This information may facilitate our understanding of how host molecules and proteins regulate microbial dynamics.

## MATERIALS AND METHODS

### Insect rearing and plants.

The whitefly *B. tabaci* MEAM1 colony (mtCO1; GenBank accession no. GQ332577) was maintained on cotton plants (Gossypium hirsutum cv. Shiyuan 321) as described previously (13, 34, 35). Cotton plants were cultivated to the 6- to 7-true-leaf stage for use in experiments.

### Symbiont elimination by antibiotic treatment.

To eliminate *Portiera*, hundreds of adult *B. tabaci* whiteflies (F0, 0 to 7 days after emergence) were released into each feeding chamber and fed on 25% sucrose solution (wt/vol) supplemented with 30 μg/mL of the antibiotic rifampin (BBI Life Sciences, Shanghai, China) for 2 days as described previously (13). Control whiteflies fed on a sucrose solution not supplemented with antibiotics. Following the antibiotic treatment, *B. tabaci* whiteflies were transferred to cotton plants. After symbiont quantification by qPCR, the *Portiera*-cured F1 *B. tabaci* (−PBt) obtained by antibiotic treatment and control *Portiera*-infected F1 *B. tabaci* (+PBt) were identified.

### qPCR and quantitative reverse transcription-PCR (qRT-PCR).

Total DNA was extracted from the whole body, bacteriocytes, and ovaries of female adult whiteflies at day 3 after the whiteflies at day 1 after emergence were microinjected with ds*Vg* in accordance with the Nonidet-P40-based protocol previously described (21). Symbionts were quantified by qPCR using the CFX96 real-time PCR detection system (Bio-Rad, Hercules, CA, USA) with 2× SYBR green master mix (Bimake, Houston, TX, USA). *Portiera* and *Hamiltonella* were quantified using symbiont-specific 16S rRNA primers, with the *B. tabaci* β-actin gene as the internal standard for normalization. All primers used are listed in Table S1 in the supplemental material. Two technical replicates were performed for each of 10 biological replicates for the whitefly whole body and for each of five biological replicates for whitefly bacteriocytes and ovaries after ds*Vg* treatment.

10.1128/mbio.02990-22.6 TABLE S1 Primers used in this study. Download Table S1, DOCX file, 0.03 MB. Copyright © 2023 Sun et al. 2023 Sun et al. https://creativecommons.org/licenses/by/4.0/ This content is distributed under the terms of the Creative Commons Attribution 4.0 International license.

Total RNA was extracted as previously described (34). To compare the expression levels of *Vg* and *VgR* in the whole body or bacteriocytes between adult females and adult males and between the whole body and bacteriocytes of adult females or adult males, RNA was extracted from the whole body of 10 female and 10 male adult whiteflies at day 1 after emergence for each of three biological replicates and from bacteriocytes of 50 female and 50 males adult whiteflies at day 1 after emergence for each of three biological replicates. To detect the effect of microinjection with pyriproxyfen or ds*JHAMT* on the expression of *Vg* in whiteflies, RNA was extracted from the whole body of 10 female adult whiteflies for each of three biological replicates at days 1 and 2 after the whiteflies at three to four days after emergence were microinjected with pyriproxyfen and from 10 female adult whiteflies for each of three biological replicates at days 5 and 6 after the whiteflies at day 1 after emergence were microinjected with ds*JHAMT*. To examine the effect of microinjection with ds*Vg* on the expression of *Vg* in whiteflies, RNA was extracted from the whole body of 10 female adult whiteflies for each of three biological replicates at days 1, 3, and 5 after the whiteflies at day 1 after emergence were microinjected with ds*Vg*. cDNAs were synthesized from the total RNA using an All-in-One cDNA synthesis supermix kit (Bimake, Houston, TX, USA) as described previously (34). The qRT-PCRs were performed using the CFX96 real-time PCR detection system (Bio-Rad, Hercules, CA, USA) with 2× SYBR green master mix (Bimake, Houston, TX, USA). Relative expression was calculated with the β-actin gene for transcript normalization. All primers used in this study are listed in Table S1. The amplification efficiency of the primers newly designed in this study is high, which is shown in Table S1. Relative symbiont density and relative gene expression were calculated using the 2^−Δ^*^CT^* method (60).

### FISH.

To investigate the localization of *Portiera* in the bacteriocytes of female adult whiteflies, FISH was conducted in accordance with a previously described protocol (13, 34, 35).

### Immunofluorescence microscopy.

Bacteriocytes and/or ovarioles were dissected from 30 female adult whiteflies at day 1 after emergence, female adult whiteflies at various days after dsRNA injection, and *Portiera*-infected and *Portiera*-cured adult female whiteflies as well as rapamycin-treated adult female whiteflies for each biological replicate. The samples were fixed, permeabilized, and incubated with Alexa Fluor 488-labeled anti-Vg (42), by following a previously described protocol (13, 34). Three biological replicates were conducted. Images were collected and analyzed using an FV3000 confocal microscope (Olympus, Tokyo, Japan). The acquisition parameters were kept constant within the experiment to allow comparison between resulting signal intensities for control/treated whitefly samples.

### Immunoelectron microscopy.

Bacteriocytes were dissected from adult females (0 to 7 days after emergence), fixed with 3% (vol/vol) paraformaldehyde and 0.2% (vol/vol) glutaraldehyde in 0.2 M phosphate buffer saline overnight at 4°C, dehydrated by a graded ethanol series (30%, 50%, 70%, 90%, and 100%), and embedded in LR Gold Resin (London Resin Company) by referring to a previously described method (36, 50, 52). Sections were cut at 80 to 100 nm with a Leica EM UC6 ultramicrotome and then blocked for 30 min in blocking buffer (goat serum, 1:100). The blocked sections were incubated at room temperature with the antibodies of anti-Vg mouse serum (1:10) for 2 h and then 10-nm gold-conjugated goat-anti-mouse IgG (1:200; IBM) for 2 h with a wash in distilled water after each antibody incubation. The sections were stained in 2% (wt/vol) neutral uranyl acetate (in distilled water) for 10 min and in lead citrate for 10 to 15 min. The sections were treated without primary antibody and without gold-conjugated goat anti-mouse IgG as the controls. Four biological replicates were conducted. The sections were viewed with a transmission electron microscope (H-7650 Hitachi) at 80-kV accelerating voltage.

### dsRNA preparation.

dsRNAs specific to whitefly *JHAMT* (ds*JHAMT*), whitefly *Vg* (ds*Vg*), and *GFP* (ds*GFP*) with sizes of 713 bp, 450 bp, and 420 bp, respectively, were synthesized using a T7 RiboMAX express RNAi system kit (Promega, USA), in accordance with the manufacturer’s instructions as described previously (13, 34, 45, 47, 57, 61).

ds*JHAMT*, ds*Vg*, and ds*GFP* correspond to nucleotide regions of 51 to 763 bp, 629 to 1,078 bp, and 76 to 495 bp (starting from the 5′ end) for the respective targets *JHAMT*, *Vg*, and *GFP* (accession numbers LOC109044195, GU332720.1, and MN623123, respectively).

### Effects of pyriproxyfen injection or silencing of *JHAMT* on *Vg* expression and Vg localization.

To study the effect of the JH pathway on *Vg* expression and Vg localization in whiteflies, approximately 800 female adult whiteflies at day 1 after emergence were injected with 1.5 μg/μL ds*JHAMT* and incubated on cotton leaf disks by using a previously described method (13, 45, 47, 57, 61). Control whiteflies were injected with 1.5 μg/μL ds*GFP*. Whiteflies were collected at 5 and 6 days after dsRNA injection. The survival rates of injected whiteflies were 60% for ds*GFP* and 40% for ds*JHAMT* at day 5 after injection. RNA was extracted from the whole body of 10 female adult whiteflies for each of three biological replicates. The expression of *JHAMT* at days 5 and 6 after ds*JHAMT* injection and the expression of *Vg* at day 5 after ds*JHAMT* injection were examined using qRT-PCR. To detect whether silencing whitefly *JHAMT* affects Vg localization in ovarioles and bacteriocytes, whiteflies were collected at day 5 after injection. For each biological replicate, ovarioles and bacteriocytes were dissected from 30 female adult whiteflies, fixed, permeabilized, and incubated with antibodies against Vg for ovarioles and bacteriocytes. The samples were incubated with no antibodies against Vg as the negative control. Three biological replicates were conducted. Images were analyzed using an FV3000 confocal microscope (Olympus, Japan). The fluorescence intensity of Vg was analyzed by Image J software. In each of three biological replicates, four ovarioles and three bacteriocytes of ds*GFP-*injected whiteflies and ds*JHAMT-*injected whiteflies were used for fluorescence intensity analysis. To further test the effect of JH on *Vg* expression, approximately 300 female adult wild-type whiteflies at 3 to 4 days after emergence were injected with 0.5 μg/μL pyriproxyfen (JH analog) dissolved in distilled water by using an Eppendorf microinjection system (Hamburg, Germany). Distilled water-injected whiteflies were used as the control. Expression of *Vg* in female adult whiteflies at days 1 and 2 after the whiteflies were microinjected with pyriproxyfen was examined using qRT-PCR with three biological replicates.

### Effects of *Portiera* elimination by antibiotic treatment on ovariole number and Vg localization in whiteflies.

*Portiera* was eliminated by antibiotic treatment as described above. Following the antibiotic treatment, *B. tabaci* whiteflies were transferred to cotton plants. F1 female adults were collected. The DNA was extracted from 12 female *B. tabaci* adults (at 3 to 7 days after eclosion) and used for symbiont quantification by qPCR. To test whether *Portiera* elimination affects the ovariole number, the number of ovarioles was scored in 10 individuals dissected in PBS at pH 7.4 for +PBt and –PBt female adult whiteflies (within 4 days after eclosion). To test whether *Portiera* elimination affects Vg localization in ovarioles and bacteriocytes, ovarioles and bacteriocytes of female *B. tabaci* adults (at 3 to 7 days after eclosion) were dissected, fixed, permeabilized, and incubated with antibodies against Vg as described above. Three biological replicates were conducted. Images were collected and analyzed on an FV3000 confocal microscope (Olympus, Japan). The fluorescence intensity of Vg was analyzed by Image J software. In each of three biological replicates, three ovarioles and bacteriocytes of +PBt and –PBt whiteflies were used for fluorescence intensity analysis.

### Effects of silencing *Vg* on Vg localization, symbiont localization and abundance, and whitefly reproduction.

To investigate whether silencing *Vg* influences Vg localization, symbiont localization and abundance, and the numbers of ovarioles and eggs, approximately 2,000 female adult whiteflies infected with *Portiera* at day 1 after emergence were injected with 1.0 μg/μL ds*Vg* in injection buffer by using an Eppendorf microinjection system (Hamburg, Germany) and incubated on cotton leaf disks as described above. Control whiteflies were injected with 1.0 μg/μL ds*GFP*. Whiteflies were collected at 3 days after dsRNA injection. The survival rates of injected whiteflies were 75% for ds*GFP* and 40% for ds*Vg* at day 3 after injection. RNA was extracted from eight female adult whiteflies for each of three biological replicates to examine the expression of *Vg* 3 days after dsRNA injection. To examine whether silencing whitefly *Vg* affects Vg localization in ovarioles and bacteriocytes and *Portiera* localization in bacteriocytes, whiteflies were collected at days 1, 3, and 5 after injection. Whitefly ovarioles and bacteriocytes were dissected, fixed, permeabilized, and incubated with antibodies against Vg for ovarioles as well as antibodies against Vg and a fluorescent probe for *Portiera* in bacteriocytes as described above. To examine whether silencing whitefly *Vg* affects *Hamiltonella* localization in bacteriocytes, whiteflies were collected at day 3 after injection. Whitefly bacteriocytes were dissected, fixed, permeabilized, and hybridized with the fluorescent probe for *Portiera* and *Hamiltonella* in bacteriocytes. Three biological replicates were conducted. Images were analyzed using a FV3000 confocal microscope (Olympus, Japan). The fluorescence intensity of Vg and *Portiera* was analyzed by Image J software. In each of three biological replicates, three ovarioles and bacteriocytes of ds*GFP-*injected whiteflies and ds*Vg-*injected whiteflies were used for fluorescence intensity analysis of Vg and three bacteriocytes of ds*GFP-*injected whiteflies and ds*Vg-*injected whiteflies were used for fluorescence intensity analysis of *Portiera*. To test whether silencing whitefly *Vg* influences the abundance of symbionts *Portiera* and *Hamiltonella*, DNA was extracted from the whole body of individual female adult whiteflies for each of 10 biological replicates and from bacteriocytes and ovaries of eight female adult whiteflies for each of five biological replicates at day 3 after the whiteflies were microinjected with ds*Vg*. Then, qPCR was performed as described above. In parallel, ovarioles were dissected in PBS at pH 7.4, and the number of ovarioles was scored in 10 individuals for ds*Vg-*injected and ds*GFP-*injected female adult whiteflies at day 3 postinjection. To determine if silencing *Vg* influences whitefly fecundity, individual *Vg-*injected and ds*GFP-*injected whiteflies were transferred onto cotton leaf disks and kept on 1.5% agar plates at 26 ± 2°C, with a 14-h-light:10-h-dark photoperiod and 60% to 80% relative humidity (RH). Egg numbers were recorded for the surviving whiteflies. Nineteen biological replicates of individuals were conducted at day 3 postinjection.

### Western blot analysis.

Whiteflies at day 1 after emergence were injected with 1.0 μg/μL ds*Vg* and 1.0 μg/μL ds*GFP* in injection buffer as described above. In total, 50 ds*GFP*-injected female adult whiteflies and 50 ds*Vg*-injected female adult whiteflies 3 days after dsRNA microinjection were collected. Proteins were extracted, separated by 12% SDS-PAGE, and then blotted by following standard procedures using previously described protocols (13, 47). Primary antibodies specific for the following proteins were used for Western blotting: Vg (42) and β-actin. The secondary antibody used was goat anti-mouse antibody for Vg (Beyotime Biotechnology, Shanghai, China). Three biological replicates were conducted. The signal was detected using a ChemiDoc XRS+ (Bio-Rad, Hercules, CA, USA). The densitometry for protein levels was analyzed using Image J software.

### IC-PCR assay.

The interaction between Vg and *Portiera* was examined using an IC-PCR assay by previously described protocols (29). PCR tubes were coated with 25 μL antibody against Vg (1:1,000 diluted in coating buffer), for 1.5 h at 37°C, and then washed five times for 5 min each time with 50 μL washing buffer. Homogenates of bacteriocytes and heads that were collected from 8 to 10 whiteflies in 5 μL PBS were incubated for 18 h at 4°C in the coated PCR tubes. The tubes were washed five times, 5 min each time, with 25 μL washing buffer and dried. PCR amplification of *Portiera* bound to the Vg protein, which was itself bound to the antibody-coated tubes, was performed with *Portiera*-specific 16S rRNA gene fragment primers (Table S1). The control no-antibody-coated tubes were incubated with a homogenate of the bacteriocytes and heads of whiteflies, and antibody-coated tubes incubated with a homogenate of whitefly heads not containing bacteriocytes served as controls.

### Statistical analyses.

For the mRNA expression level, fluorescence intensity, protein level, and symbiont abundance, as well as the numbers of Vg gold dots, ovarioles, and eggs, statistical differences were evaluated using one-way analysis of variance (ANOVA) at a significance threshold of 0.05. All of the data analyses were conducted using STATISTICA v6.1 software (StatSoft, Inc., Tulsa, OK, USA).

### Ethical approval and consent to participate.

The insects used in this study are agricultural pests. No ethics approval is needed.

### Data availability.

All data generated or analyzed during this study are included in the article and the supplemental material.

## References

[1] Moran NA, McCutcheon JP, Nakabachi A. 2008. Genomics and evolution of heritable bacterial symbionts. Annu Rev Genet 42:165–190. doi:10.1146/annurev.genet.41.110306.130119. 18983256

[2] Douglas AE. 2015. Multiorganismal insects: diversity and function of resident microorganisms. Annu Rev Entomol 60:17–34. doi:10.1146/annurev-ento-010814-020822. 25341109 PMC4465791

[3] Engelstädter J, Hurst GD. 2009. The ecology and evolution of microbes that manipulate host reproduction. Annu Rev Ecol Evol Syst 40:127–149. doi:10.1146/annurev.ecolsys.110308.120206.

[4] Ma WJ, Schwander T. 2017. Patterns and mechanisms in instances of endosymbiont-induced parthenogenesis. J Evol Biol 30:868–888. doi:10.1111/jeb.13069. 28299861

[5] Bondy EC, Hunter MS. 2019. Sex ratios in the haplodiploid herbivores, Aleyrodidae and Thysanoptera: a review and tools for study. Adv Insect Physiol 56:251–281. doi:10.1016/bs.aiip.2019.01.002.

[6] Perlmutter JI, Bordenstein SR. 2020. Microorganisms in the reproductive tissues of arthropods. Nat Rev Microbiol 18:97–111. doi:10.1038/s41579-019-0309-z. 31907461 PMC8022352

[7] Hosokawa T, Koga R, Kikuchi Y, Meng XY, Fukatsu T. 2010. *Wolbachia* as a bacteriocyte-associated nutritional mutualist. Proc Natl Acad Sci USA 107:769–774. doi:10.1073/pnas.0911476107. 20080750 PMC2818902

[8] Nikoh N, Hosokawa T, Moriyama M, Oshima K, Hattori M, Fukatsu T. 2014. Evolutionary origin of insect-*Wolbachia* nutritional mutualism. Proc Natl Acad Sci USA 111:10257–10262. doi:10.1073/pnas.1409284111. 24982177 PMC4104916

[9] Michalkova V, Benoit JB, Weiss BL, Attardo GM, Aksoy S. 2014. Vitamin B6 generated by obligate symbionts is critical for maintaining proline homeostasis and fecundity in tsetse flies. Appl Environ Microbiol 80:5844–5853. doi:10.1128/AEM.01150-14. 25038091 PMC4178588

[10] Moriyama M, Nikoh N, Hosokawa T, Fukatsu T. 2015. Riboflavin provisioning underlies *Wolbachia*’s fitness contribution to its insect host. mBio 6:e01732-15. doi:10.1128/mBio.01732-15. 26556278 PMC4659472

[11] Snyder AK, Rio RV. 2015. “*Wigglesworthia morsitans*” folate (vitamin B9) biosynthesis contributes to tsetse host fitness. Appl Environ Microbiol 81:5375–5386. doi:10.1128/AEM.00553-15. 26025907 PMC4510189

[12] Ju JF, Bing XL, Zhao DS, Guo Y, Xi Z, Hoffmann AA, Zhang KJ, Huang HJ, Gong JT, Zhang X, Hong XY. 2020. *Wolbachia* supplement biotin and riboflavin to enhance reproduction in planthoppers. ISME J 14:676–687. doi:10.1038/s41396-019-0559-9. 31767943 PMC7031331

[13] Ren FR, Sun X, Wang TY, Yan JY, Yao YL, Li CQ, Luan JB. 2021. Pantothenate mediates the coordination of whitefly and symbiont fitness. ISME J 15:1655–1667. doi:10.1038/s41396-020-00877-8. 33432136 PMC8163847

[14] Hunter MS, Perlman SJ, Kelly SE. 2003. A bacterial symbiont in the *Bacteroidetes* induces cytoplasmic incompatibility in the parasitoid wasp *Encarsia pergandiella*. Proc Biol Sci 270:2185–2190. doi:10.1098/rspb.2003.2475. 14561283 PMC1691482

[15] Beckmann JF, Ronau JA, Hochstrasser M. 2017. A *Wolbachia* deubiquitylating enzyme induces cytoplasmic incompatibility. Nat Microbiol 2:17007. doi:10.1038/nmicrobiol.2017.7. 28248294 PMC5336136

[16] Harumoto T, Lemaitre B. 2018. Male-killing toxin in a *Drosophila* bacterial symbiont. Nature 557:252–255. doi:10.1038/s41586-018-0086-2. 29720654 PMC5969570

[17] Serbus LR, Casper-Lindley C, Landmann F, Sullivan W. 2008. The genetics and cell biology of *Wolbachia*-host interactions. Annu Rev Genet 42:683–707. doi:10.1146/annurev.genet.41.110306.130354. 18713031

[18] Tufail M, Takeda M. 2008. Molecular characteristics of insect vitellogenins. J Insect Physiol 54:1447–1458. doi:10.1016/j.jinsphys.2008.08.007. 18789336

[19] Raikhel AS, Dhadialla TS. 1992. Accumulation of yolk proteins in insect oocytes. Annu Rev Entomol 37:217–251. doi:10.1146/annurev.en.37.010192.001245. 1311540

[20] Sappington TW, Raikhel AS. 1998. Molecular characteristics of insect vitellogenins and vitellogenin receptors. Insect Biochem Mol Biol 28:277–300. doi:10.1016/s0965-1748(97)00110-0. 9692232

[21] Luan J, Sun X, Fei Z, Douglas AE. 2018. Maternal inheritance of a single somatic animal cell displayed by the bacteriocyte in the whitefly *Bemisia tabaci*. Curr Biol 28:459–465.e3. doi:10.1016/j.cub.2017.12.041. 29395925 PMC5807091

[22] Fukatsu T. 2021. The long and winding road for symbiont and yolk protein to host oocyte. mBio 12:e02997-20. doi:10.1128/mBio.02997-20. PMC7885100 33563820

[23] Koga R, Meng XY, Tsuchida T, Fukatsu T. 2012. Cellular mechanism for selective vertical transmission of an obligate insect symbiont at the bacteriocyte-embryo interface. Proc Natl Acad Sci USA 109:e1230-7–E1237. doi:10.1073/pnas.1119212109. 22517738 PMC3356617

[24] Herren JK, Paredes JC, Schüpfer F, Lemaitre B. 2013. Vertical transmission of a *Drosophila* endosymbiont via cooption of the yolk transport and internalization machinery. mBio 4:e00532-12. doi:10.1128/mBio.00532-12. 23462112 PMC3585447

[25] Li Z, Zhang S, Zhang J, Liu M, Liu Z. 2009. Vitellogenin is a cidal factor capable of killing bacteria via interaction with lipopolysaccharide and lipoteichoic acid. Mol Immunol 46:3232–3239. doi:10.1016/j.molimm.2009.08.006. 19729202

[26] Salmela H, Amdam GV, Freitak D. 2015. Transfer of immunity from mother to offspring is mediated via egg-yolk protein vitellogenin. PLoS Pathog 11:e1005015. doi:10.1371/journal.ppat.1005015. 26230630 PMC4521805

[27] Park HG, Lee KS, Kim BY, Yoon HJ, Choi YS, Lee KY, Wan H, Li J, Jin BR. 2018. Honeybee (*Apis cerana*) vitellogenin acts as an antimicrobial and antioxidant agent in the body and venom. Dev Comp Immunol 85:51–60. doi:10.1016/j.dci.2018.04.001. 29621531

[28] Guo Y, Hoffmann AA, Xu XQ, Mo PW, Huang HJ, Gong JT, Ju JF, Hong XY. 2018. Vertical transmission of *Wolbachia* is associated with host vitellogenin in *Laodelphax striatellus*. Front Microbiol 9:2016. doi:10.3389/fmicb.2018.02016. 30233514 PMC6127624

[29] Brumin M, Lebedev G, Kontsedalov S, Ghanim M. 2020. Levels of the endosymbiont *Rickettsia* in the whitefly *Bemisia tabaci* are influenced by the expression of vitellogenin. Insect Mol Biol 29:241–255. doi:10.1111/imb.12629. 31825546

[30] De Barro PJ, Liu SS, Boykin LM, Dinsdale AB. 2011. *Bemisia tabaci*: a statement of species status. Annu Rev Entomol 56:1–19. doi:10.1146/annurev-ento-112408-085504. 20690829

[31] Liu SS, De Barro PJ, Xu J, Luan JB, Zang LS, Ruan YM, Wan FH. 2007. Asymmetric mating interactions drive widespread invasion and displacement in a whitefly. Science 318:1769–1772. doi:10.1126/science.1149887. 17991828

[32] Gottlieb Y, Ghanim M, Gueguen G, Kontsedalov S, Vavre F, Fleury F, Zchori-Fein E. 2008. Inherited intracellular ecosystem: symbiotic bacteria share bacteriocytes in whiteflies. FASEB J 22:2591–2599. doi:10.1096/fj.07-101162. 18285399

[33] Skaljac M, Zanic K, Ban SG, Kontsedalov S, Ghanim M. 2010. Co-infection and localization of secondary symbionts in two whitefly species. BMC Microbiol 10:142. doi:10.1186/1471-2180-10-142. 20462452 PMC2877686

[34] Ren FR, Sun X, Wang TY, Yao YL, Huang YZ, Zhang X, Luan JB. 2020. Biotin provisioning by horizontally transferred genes from bacteria confers animal fitness benefits. ISME J 14:2542–2553. doi:10.1038/s41396-020-0704-5. 32572143 PMC7490365

[35] Wang YB, Ren FR, Yao YL, Sun X, Walling LL, Li NN, Bai B, Bao XY, Xu XR, Luan JB. 2020. Intracellular symbionts drive sex ratio in the whitefly by facilitating fertilization and provisioning of B vitamins. ISME J 14:2923–2935. doi:10.1038/s41396-020-0717-0. 32690936 PMC7784916

[36] Luan JB, Shan HW, Isermann P, Huang JH, Lammerding J, Liu SS, Douglas AE. 2016. Cellular and molecular remodelling of a host cell for vertical transmission of bacterial symbionts. Proc Biol Sci 283:20160580. doi:10.1098/rspb.2016.0580. 27358364 PMC4936034

[37] Sloan DB, Moran NA. 2012. Endosymbiotic bacteria as a source of carotenoids in whiteflies. Biol Lett 8:986–989. doi:10.1098/rsbl.2012.0664. 22977066 PMC3497135

[38] Chen W, Hasegawa DK, Kaur N, Kliot A, Pinheiro PV, Luan J, Stensmyr MC, Zheng Y, Liu W, Sun H, Xu Y, Luo Y, Kruse A, Yang X, Kontsedalov S, Lebedev G, Fisher TW, Nelson DR, Hunter WB, Brown JK, Jander G, Cilia M, Douglas AE, Ghanim M, Simmons AM, Wintermantel WM, Ling KS, Fei Z. 2016. The draft genome of whitefly *Bemisia tabaci* MEAM1, a global crop pest, provides novel insights into virus transmission, host adaptation, and insecticide resistance. BMC Biol 14:110. doi:10.1186/s12915-016-0321-y. 27974049 PMC5157087

[39] Bell WJ, Barth RH. 1971. Initiation of yolk deposition by juvenile hormone. Nat New Biol 230:220–222. doi:10.1038/newbio230220a0. 5280174

[40] Santos CG, Humann FC, Hartfelder K. 2019. Juvenile hormone signaling in insect oogenesis. Curr Opin Insect Sci 31:43–48. doi:10.1016/j.cois.2018.07.010. 31109672

[41] Tufail M, Nagaba Y, Elgendy AM, Takeda M. 2014. Regulation of vitellogenin genes in insects. Entomol Sci 17:269–282. doi:10.1111/ens.12086.

[42] Guo JY, Dong SZ, Yang XL, Cheng L, Wan FH, Liu SS, Zhou XP, Ye GY. 2012. Enhanced vitellogenesis in a whitefly via feeding on a begomovirus-infected plant. PLoS One 7:e43567. doi:10.1371/journal.pone.0043567. 22937062 PMC3427354

[43] Li A, Sadasivam M, Ding JL. 2003. Receptor-ligand interaction between vitellogenin receptor (VtgR) and vitellogenin (Vtg), implications on low density lipoprotein receptor and apolipoprotein B/E. J Biol Chem 278:2799–2806. doi:10.1074/jbc.M205067200. 12429745

[44] Tufail M, Takeda M. 2009. Insect vitellogenin/lipophorin receptors: molecular structures, role in oogenesis, and regulatory mechanisms. J Insect Physiol 55:87–103. doi:10.1016/j.jinsphys.2008.11.007. 19071131

[45] Li NN, Jiang S, Lu KY, Hong JS, Wang YB, Yan JY, Luan JB. 2022. Bacteriocyte development is sexually differentiated in *Bemisia tabaci*. Cell Rep 38:110613. doi:10.1016/j.celrep.2022.110613. 35354051

[46] Bellés X, Martín D, Piulachs MD. 2005. The mevalonate pathway and the synthesis of juvenile hormone in insects. Annu Rev Entomol 50:181–199. doi:10.1146/annurev.ento.50.071803.130356. 15355237

[47] Bao XY, Yan JY, Yao YL, Wang YB, Visendi P, Seal S, Luan JB. 2021. Lysine provisioning by horizontally acquired genes promotes mutual dependence between whitefly and two intracellular symbionts. PLoS Pathog 17:e1010120. doi:10.1371/journal.ppat.1010120. 34843593 PMC8659303

[48] Moran NA, Bennett GM. 2014. The tiniest tiny genomes. Annu Rev Microbiol 68:195–215. doi:10.1146/annurev-micro-091213-112901. 24995872

[49] Wilson ACC, Duncan RP. 2015. Signatures of host/symbiont genome coevolution in insect nutritional endosymbioses. Proc Natl Acad Sci USA 112:10255–10261. doi:10.1073/pnas.1423305112. 26039986 PMC4547219

[50] Huo Y, Liu W, Zhang F, Chen X, Li L, Liu Q, Zhou Y, Wei T, Fang R, Wang X. 2014. Transovarial transmission of a plant virus is mediated by vitellogenin of its insect vector. PLoS Pathog 10:e1003949. doi:10.1371/journal.ppat.1003949. 24603905 PMC3946389

[51] Wei J, He YZ, Guo Q, Guo T, Liu YQ, Zhou XP, Liu SS, Wang XW. 2017. Vector development and vitellogenin determine the transovarial transmission of begomoviruses. Proc Natl Acad Sci USA 14:6746–6751. 10.1073/pnas.1701720114 PMC5495249 28607073

[52] Mao Q, Wu W, Huang L, Yi G, Jia D, Chen Q, Chen H, Wei T. 2020. Insect bacterial symbionts-mediated vitellogenins uptake into oocytes to support egg development. mBio 11:e01142-20. doi:10.1128/mBio.01142-20. 33172995 PMC7667026

[53] Li Z, Zhang S, Liu Q. 2008. Vitellogenin functions as a multivalent pattern recognition receptor with an opsonic activity. PLoS One 3:e1940. doi:10.1371/journal.pone.0001940. 18398466 PMC2277463

[54] Havukainen H, Münch D, Baumann A, Zhong S, Halskau Ø, Krogsgaard M, Amdam GV. 2013. Vitellogenin recognizes cell damage through membrane binding and shields living cells from reactive oxygen species. J Biol Chem 288:28369–28381. doi:10.1074/jbc.M113.465021. 23897804 PMC3784755

[55] Liu QH, Zhang SC, Li ZJ, Gao CR. 2009. Characterization of a pattern recognition molecule vitellogenin from carp (*Cyprinus carpio*). Immunobiology 214:257–267. doi:10.1016/j.imbio.2008.10.003. 19327543

[56] Costa HS, Westcot DM, Ullman DE, Johnson MW. 1993. Ultrastructure of the endosymbionts of the whitefly, *Bemisia tabaci* and *Trialeurodes vaporariorum*. Protoplasma 176:106–115. doi:10.1007/BF01378946.

[57] Wang YB, Li C, Yan JY, Wang TY, Yao YL, Ren FR, Luan JB. 2022. Autophagy regulates whitefly-symbiont metabolic interactions. Appl Environ Microbiol 88:e0208921. doi:10.1128/AEM.02089-21. 34818107 PMC8824202

[58] Nozaki T, Shigenobu S. 2022. Ploidy dynamics in aphid host cells harboring bacterial symbionts. Sci Rep 12:9111. doi:10.1038/s41598-022-12836-8. 35650254 PMC9159990

[59] James EB, Pan X, Schwartz O, Wilson ACC. 2022. SymbiQuant: a machine learning object detection tool for polyploid independent estimates of endosymbiont population size. Front Microbiol 13:816608. doi:10.3389/fmicb.2022.816608. 35663891 PMC9160162

[60] Schmittgen TD, Livak KJ. 2008. Analyzing real-time PCR data by the comparative C(T) method. Nat Protoc 3:1101–1108. doi:10.1038/nprot.2008.73. 18546601

[61] Sun X, Liu BQ, Li CQ, Chen ZB, Xu XR, Luan JB. 2022. A novel microRNA regulates cooperation between symbionts and a laterally acquired gene in the regulation of pantothenate biosynthesis within *Bemisia tabaci* whiteflies. Mol Ecol 31:2611–2624. doi:10.1111/mec.16416. 35243711

