# Supplementary figures and images for: Vitellogenin Facilitates Associations between the Whitefly and a Bacteriocyte Symbiont
Source: mBio. 2023 Jan 24;14(1):e02990-22. doi: 10.1128/mbio.02990-22 (PMC9973357; doi:10.1128/mbio.02990-22)

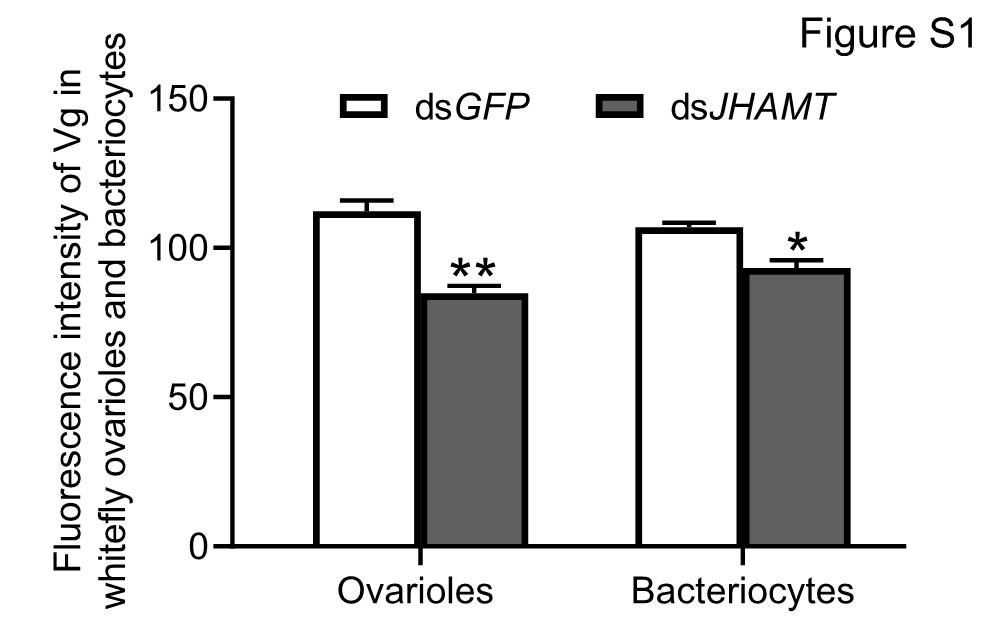

Supplement: FIG S1 [file mbio.02990-22-s0001.tif]

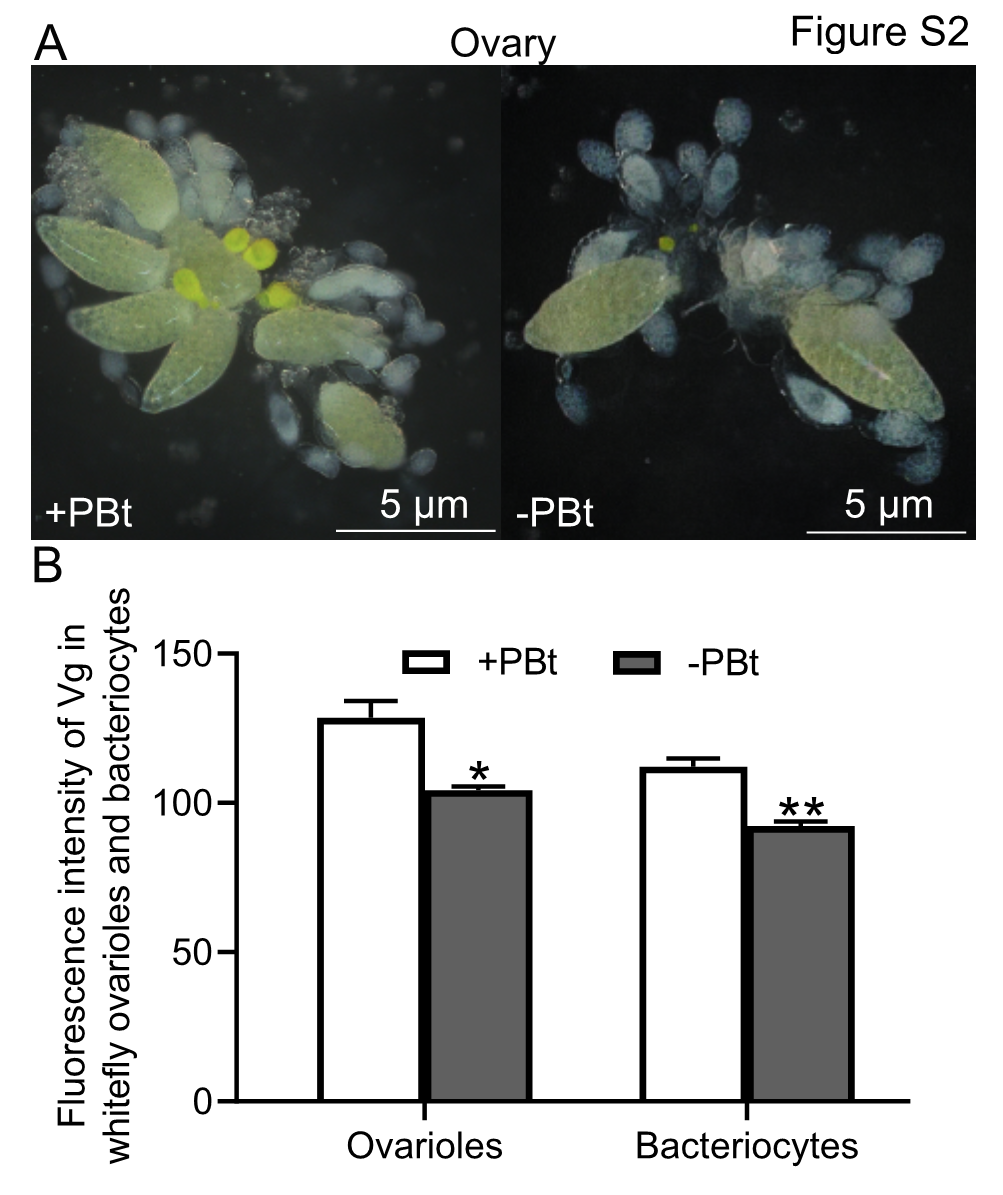

Supplement: FIG S2 [file mbio.02990-22-s0002.tif]

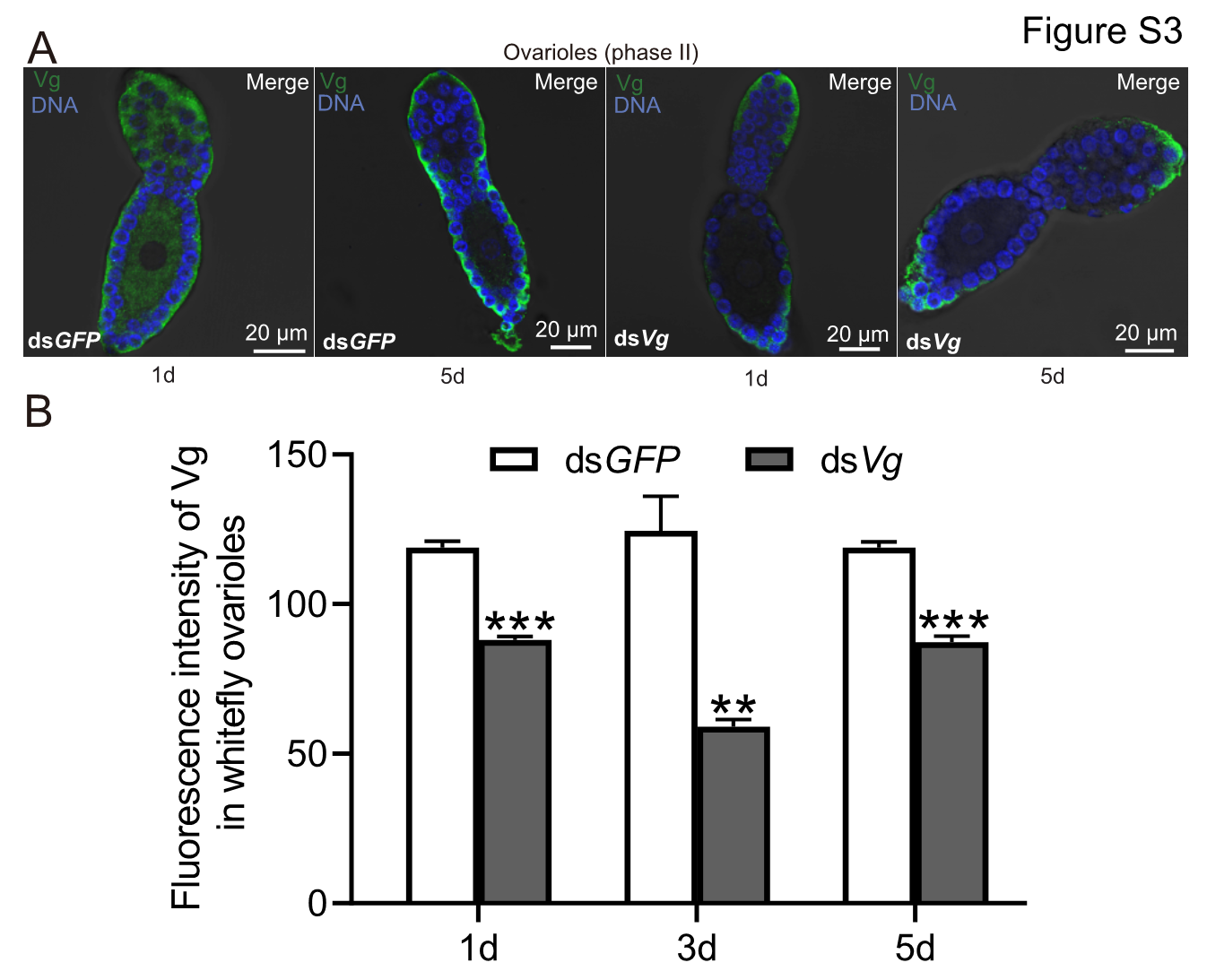

Supplement: FIG S3 [file mbio.02990-22-s0003.tif]

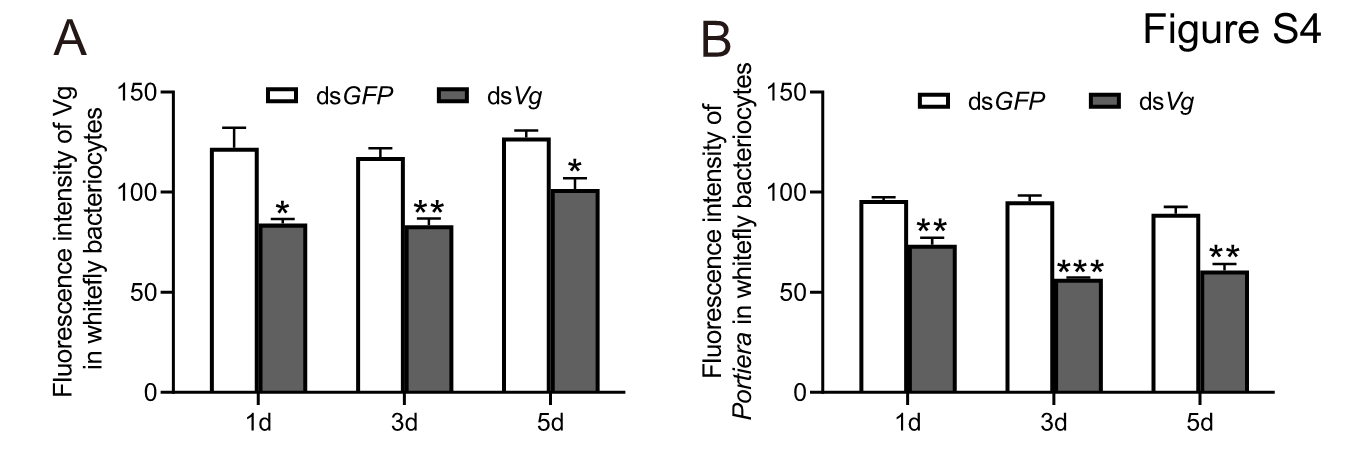

Supplement: FIG S4 [file mbio.02990-22-s0004.tif]

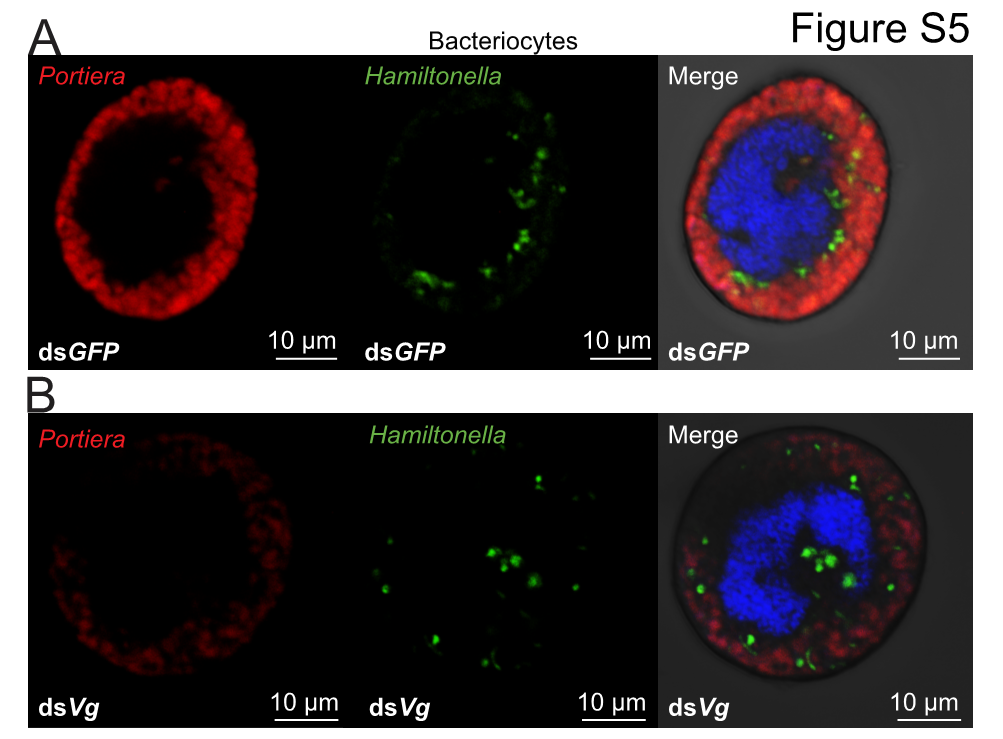

Supplement: FIG S5 [file mbio.02990-22-s0005.tif]
