# Supplementary material for: Vitellogenin Facilitates Associations between the Whitefly and a Bacteriocyte Symbiont
Source: mBio. 2023 Jan 24;14(1):e02990-22. doi: 10.1128/mbio.02990-22 (PMC9973357; doi:10.1128/mbio.02990-22)
Supplement: TABLE S1 [file mbio.02990-22-s0006.docx]

**Table S1 Primer sequence (Related to Fig. 1-4)**

| *Gene name* | *Application* | | *5'-3'* | *Ampliﬁcation efﬁciency (%)* |
| --- | --- | --- | --- | --- |
| (a) Quantitative reverse transcription PCR (qRT-PCR) | | | |  |
| *Vg*-F^1^ | Gene expression | | ACAAGTCTCCGACGCCGAAG |  |
| *Vg*-R^1^ |  | | TTGACATCGGCTTTACGGCA |  |
| *VgR-F* | Gene expression | | CGATGCCAAGGATAGATG | 101.34 |
| *VgR-R* |  | | CGAAACTCGCTGGAAGG |  |
| *JHAMT*-F | Gene expression | | AGATGTGGCGAACCTTCTGA | 100.92 |
| *JHAMT*-R |  | | CAGAACCCTTTGGTTTGGCA |  |
| *qRT-actin*-F^2^ | Reference gene | | TGGAGATGGTGTTTCCCACAC |  |
| *qRT-actin*-R^2^ |  | | CCAGCCAAGTCCAAACGAAG |  |
| (b) Quantitative PCR (qPCR) | | | |  |
| H-16S-*F*^3^ | *Hamiltonella* density | | GCATCGAGTGAGCACAGTTT |  |
| H-16S-*R*^3^ |  | | TATCCTCTCAGACCCGCTAGA |  |
| Port73/Port266-*F*^4^ | *Portiera* density | | GTGGGGAATAACGTACGG |  |
| Port73/Port266-*R*^4^ |  | | CTCAGTCCCAGTGTGGCTG |  |
| glt375-*F*^4^ | *Rickettsia* density | | TGGTATTGCATCGCTTTGGG |  |
| glt375-*R*^4^ |  | | TTTCTTTAAGCACTGCAGCACG |  |
| q-actin-*F*^5^ | Reference gene | | TCTTCCAGCCATCCTTCTTG |  |
| q-actin-*R*^5^ |  | | CGGTGATTTCCTTCTGCATT |  |
| (c) PCR | | | |  |
| Por-F^6^ | *Portiera* detection | | GGAAACGTACGCTAATAC |  |
| Por-R^6^ |  | | TGACGACAGCCATGCAGCAC |  |
| (d) dsRNA synthesis | |  | |  |
| ds*JHAMT*-F | Gene silencing | GGATCCTAATACGACTCACTATAGGGTGTGGCGAACCTTCTGAA | |  |
| ds*JHAMT*-R |  | GGATCCTAATACGACTCACTATAGGGGGTATGGACGGCTTGGTTA | |  |
| *dsVg*-F | Gene silencing | GGATCCTAATACGACTCACTATAGGGACATCGTCAAGGCCACCAA | |  |
| *dsVg*-R |  | GGATCCTAATACGACTCACTATAGGGTAGAGCTGGAACTAGATGAG | |  |
| *dsGFP*-F | Gene silencing | GGATCCTAATACGACTCACTATAGGGCACAAGTTCAGCGTGTCCG | |  |
| *dsGFP*-R |  | GGATCCTAATACGACTCACTATAGGGGTTCACCTTGATGCCGTTC | |  |

**SI References**

1. Wei J, He YZ, Guo Q, Guo T, Liu YQ, Zhou XP, Liu SS, Wang XW. 2017. Vector development and vitellogenin determine the transovarial transmission of begomoviruses. Proc Natl Acad Sci U S A 114:6746-6751.
2. Wang ZZ, Shi M, Ye XQ, Chen MY, Chen XX. 2013. Identification, characterization and expression of a defensin-like antifungal peptide from the whitefly *Bemisia tabaci* (gennadius) (hemiptera: aleyrodidae). Insect Mol. Biol 22:297-305.
3. Brumin M, Kontsedalov S, Ghanim M. 2011. *Rickettsia* influences thermotolerance in the whitefly *Bemisia tabaci* B biotype. Insect Sci 18:57-66.
4. Caspi-Fluger A, Inbar, M, Mozes-Daube N, Mouton.L, Hunter MS, Zchori-Fein E. 2011. *Rickettsia* ‘in’ and ‘out’: two different localization patterns of a bacterial symbiont in the same insect species. PLoS One 6: e21096.
5. Sinisterra XH., McKenzie CL, Hunter WB, Powell CA, Shatters RG. 2005. Differential transcriptional activity of plant-pathogenic begomoviruses in their whitefly vector (*Bemisia tabaci*, Gennadius: Hemiptera Aleyrodidae). J Gen Virol 86:1525-1532.
6. Thierry M, Becker N, Hajri A, Reynaud B, Lett JM, Delatte H. 2011. Symbiont diversity and non-random hybridization among indigenous (Ms) and invasive (B) biotypes of *Bemisia tabaci*. Mol Ecol 20:172-2187.
